# Tuberculosis prevention, diagnosis, and treatment financial profile during 2006–2021: PART A

**DOI:** 10.1186/s12962-023-00479-z

**Published:** 2023-09-19

**Authors:** Maryam Meskini, Nahid Madadi, Kamal Ahmadi, Farzam Vaziri, Abolfazl Fateh, Seyed Davar Siadat

**Affiliations:** 1 Department of Mycobacteriology and Pulmonary Research, Pasteur Institute of Iran, Tehran, Iran; 2 Microbiology Research Center (MRC), Pasteur Institute of Iran, Tehran, Iran

**Keywords:** Tuberculosis, Prevention, Diagnosis, Treatment, And Financial Profile

## Abstract

**Background:**

Tuberculosis (TB) is a communicable disease that is a major cause of death and one of the leading causes of death worldwide. Currently, there is no analyzed data to examine the financial profile of TB by country, continent, and year; this article analyzed TB prevention, diagnosis, and treatment financial profile during the last two decades.

**Methods:**

Original research, reviews, and governmental databases are analyzed to present the financial profile of TB.

**Results:**

Analyzed data showed Europe (23137.133), Asia (20137.073), and Africa (15237.973) had the most allocated funds (US $ million), and Oceania (236.702), and America (4745.043) had the lowest allocated fund (US $ million) during 2006–2021. Additionally, the allocation of funds (domestic funds, global funds, and grants [excluding global funds]) in different countries and proper planning for TB eradication has caused that in the last two decades, the slope of the confirmed cases and deaths graph line is negative.

**Conclusion:**

The number of confirmed cases and deaths reported globally is decreasing. The trend lines showed that the assigned funds are increasing, indicating that the TB eradication plan can be apprehended soon.

**Supplementary Information:**

The online version contains supplementary material available at 10.1186/s12962-023-00479-z.

## Introduction

Tuberculosis (TB) is a global infectious disease, and its morbidity and mortality remain one of the major global health challenges. Till the coronavirus disease 2019 (COVID-19) pandemic, TB was the leading cause of death from a single infectious agent, ranking above human immunodeficiency virus (HIV)/ acquired immunodeficiency syndrome (AIDS). The number of people newly diagnosed reported with TB was 7.1 and 5.8 million in 2019 and 2020, respectively [1]. Tuberculosis is reported as one of the ten most important causes of death from infectious diseases worldwide [2, 3]. About one-third of the world’s population is infected by *Mycobacterium tuberculosis* [4]. The World Health Organization (WHO) reported 5.8 million people suffering from TB, and 1.51 million (1.3 million HIV-negative, 0.21 HIV-positive) people died in 2021 [5]. More than 95% of TB deaths occur in developing countries [6]. Factors such as immune system deficiency, the prevalence of multi-drug resistant strains of *M. tuberculosis*, and people’s aging are considered risk factors for contracting TB [7, 8]. Despite many scientific advances and the efforts of various international organizations, TB is still one of the most life-threatening diseases in the world [9, 10]. Geographically, most cases of TB are in Southeast Asia, Africa, and the West Pacific respectively [11]. In case of no appropriate treatment for TB and an increase in drug resistance, treatment procedures of patients face serious problems and lead to increased costs of treatment and side effects. Multi-drug-resistant TB (MDR-TB) can be present in primary TB infection or be considered a complication during treatment. In 2018, 3.4% of newly diagnosed TB patients and 18% of previously treated TB patients worldwide were infected by MDR-TB strains [12]. TB and its complications cause many economic problems. Despite the fact that TB diagnosis and treatment processes are free, TB patients incur a lot of costs. The expensive care and treatment of TB can lead to a delay in diagnosis and, as a result, inappropriate treatment of patients, which leads to the increase the rate of MDR strains that require more expensive treatment [13, 14].

According to the WHO report in 2020, out of the estimated cost of 5.6 billion dollars, 4.2 billion and 2.26 billion were allocated for diagnosing and treating drug-sensitive and drug-resistant TB, respectively. The rest of the remaining funds were considered for cases such as TB preventive treatment, specific interventions for HIV-related TB, and miscellaneous items [15]. The High-Level Panel for the United Nations Sustainable Development Goals (SDGs) has estimated that an investment of US$1 in TB care brings a return of US$ 30. Other studies have estimated this return to be as high as US$ 115 for every dollar invested in TB care [16]. Hence, TB preventions, diagnosis, and treatment financial profile analyzing is very important due to the significant and especial condition of TB. This analyzing information help authorities and government to decide in which country or continent to spend TB funds that can contribute to the TB eradication program. To the best of our knowledge, this is the first analysis paper that analyzes TB prevention, diagnosis, and treatment financial profile during 2006–2021 based on the WHO annual report.

## Methods

We considered data reported by countries to the WHO and estimates of the TB burden generated by the WHO for the Global Tuberculosis Report from 2006 to 2021 (https://www.who.int/teams/global-tuberculosis-programme/data).

In the Financing for TB prevention, diagnosis, and treatment data (https://app.powerbi.com/view?r=eyJrIjoiMGIwZDUzMmItODE5Yi00YjAzLTliMGEtNGVhMGVlYzA4YWVkIiwidCI6ImY2MTBjMGI3LWJkMjQtNGIzOS04MTBiLTNkYzI4MGFmYjU5MCIsImMiOjh9), we address data of funding by source (US$ million). Next, extracted all data by continent, country, and years (2006–2021) were categorized into three groups follow as domestic, global fund, and grants (excluding global fund) (Supplementary Table 1). Subsequently, all data were analyzed by GraphPad Prism version 9.3 (prism.exe).

## Results and discussion

### Tuberculosis and world health organization plan

A global milestone for reducing the burden of TB (defined as TB incidence and TB deaths) by all WHO and United Nations) UN (Member States through the adoption of the WHO End TB Strategy (2016–2035) in the World Health Assembly (WHA) in 2014 and the Sustainable Development Goals (SDGs) in the UN General Assembly in 2015 [17].

The first milestone of the end TB strategy is a 20% reduction in TB incidence rates (the number of new and relapsed cases per 100,000 people per year) by 2020 compared to 2015. The second milestone in 2025 is a 50% reduction compared to 2015, followed by 80% reduction targets by 2030 and a 90% reduction by 2035. Tuberculosis incidence per 100,000 people per year as the indicator to measure progress toward ending the global TB epidemic by 2030 is an important target of SDG 3 [18]. Achieving the milestones and targets requires an annual reduction in TB incidence rates of 4–5% by 2020, accelerating to 10% per year by 2025 and then an average of 17% per year from 2025 to 2035. Once the milestones and targets were determined, the key requirements to achieve them were identified. They included the provision of TB prevention, diagnosis, and treatment services in the context of progress toward universal health coverage (UHC), multisectoral actions to address the various social and economic determinants of TB, and technological advances (such as a new vaccine by 2025) [19, 20].

### Tuberculosis financial profile by year

Progress in reducing the burden of TB requires sufficient funding to be sustained over many years and accessible to all countries. To this end, the WHO began annual monitoring of funding for TB prevention, diagnosis, and treatment services in 2002. Findings have been published in global TB reports and peer-reviewed journals [21–23]. This funding source includes domestic, global, and grants (excluding global funds). The detail of this funding is provided in Supplementary Table 1.

**Fig. 1:**
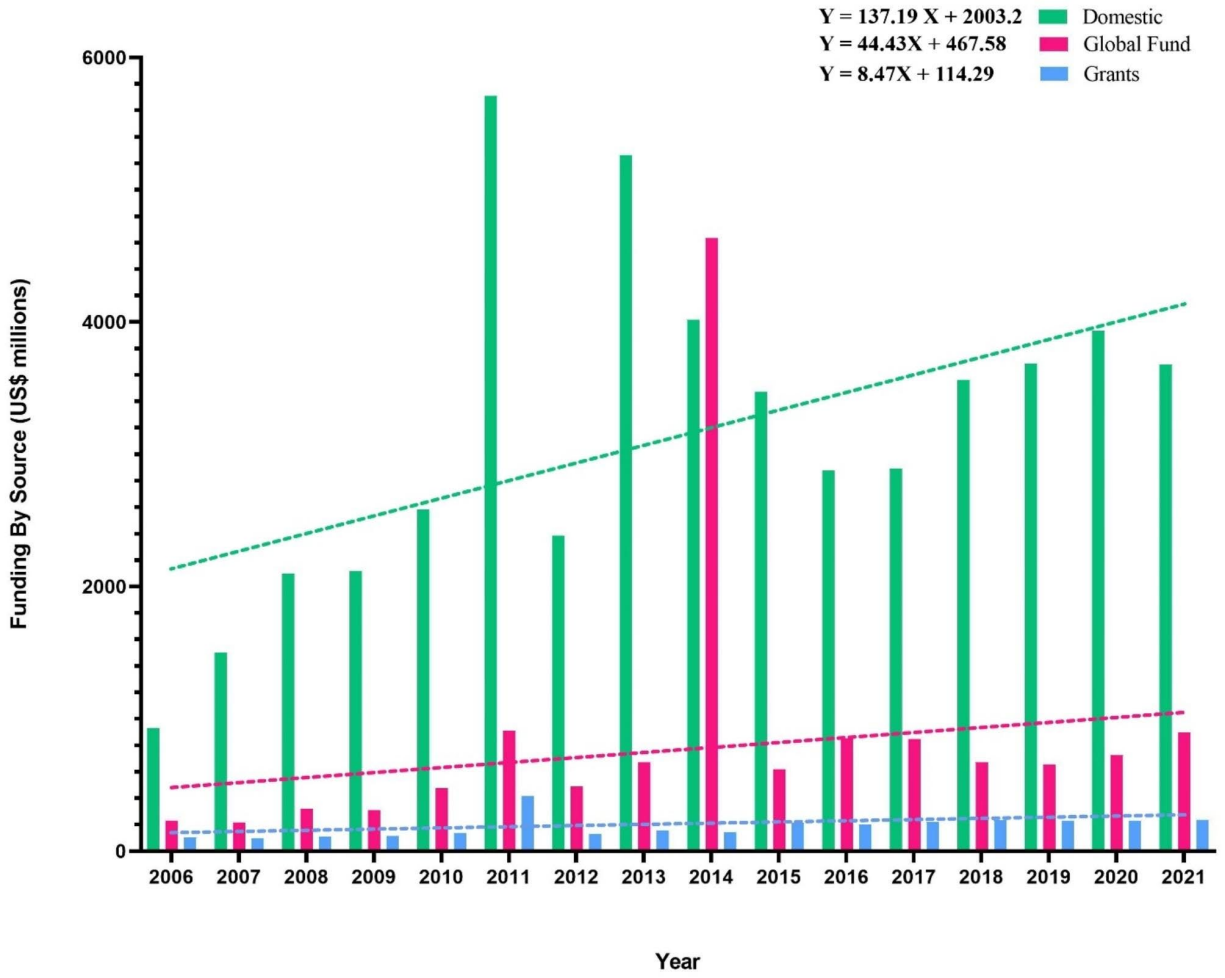
Analysis of funding by source (US $ million) per year from 2006-2021base on the global TB report 2021

The updated data from financial for TB prevention, diagnosis, and treatment worldwide can be found on the WHO website. Based on our analysis, the domestic fund is 3.75 times more than the global fund and 17 times more than the grant (excluding the global fund) during 2006–2021. Furthermore, the global fund is 4.54 times more than grants. Correspondingly, the trend lines and positive formula slopes showed that the whole source funding is increasing (Fig. 1). Based on the formulas, the slope of the domestic fund formula is 137.19, which is higher than the global fund and grants (Fig. 1), that is indicated that governments have planned for the TB eradication plan identified by the WHO and have taken steps to achieve the set goals.

On the other hand, domestic funds in 2011, 2013, and 2020 had the most budget during 2006–2021. In 2014 global funds and 2011 grants had the most budget worldwide during 2006–2021. In total, 2014, 2011, and 2013, respectively, have allocated the most funds globally between the years 2006 to 2021. These results may correlate with the decreased slope formula of confirmed and death cases from 2006 to 2020, which is confirmed by the eradication of TB that the WHO indicates. Moreover, the least allocated funds were in 2006, 2007, and 2008. It specifies that fewer funds were allocated to this plan in the early years of the TB eradication plan and increased accordingly.

**Fig. 2:**
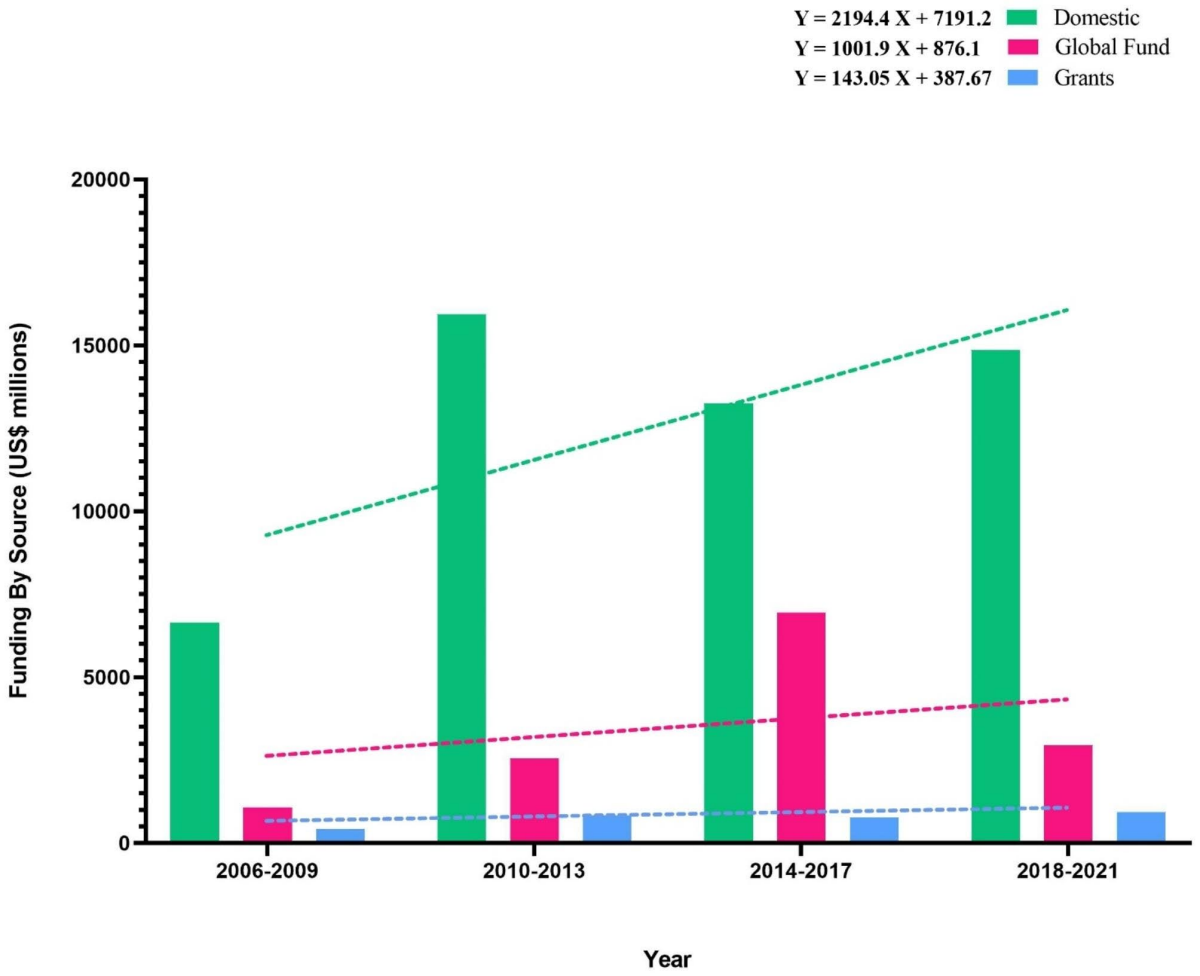
Analysis of funding by source (US $ million) in 4-year intervals from 2006-2021base on the global TB report 2021

In this part, we categorized the data into four groups, 2006–2009, 2010–2013, 2014–2017, and 2018–2022. Figure 2 illustrates that the domestic fund has the most budget in the second four years (2010–2013). The global fund has the most budget in the third group (2014–2017). The grant in 2018–2021 has the most budget during the last two decades. Our analysis showed that the most budget in the last two decades had been allocated from 2014 to 2017.

Figure 2 indicates that the trend lines and positive formula slopes in whole source funding are increasing. Based on the formulas, the slope of the domestic fund formula is 2194.4, which is higher than the global fund and grants (Fig. 2). The slope formula of the domestic fund is 2.1 times more than the global fund, followed by 15.34 times more than grants. Furthermore, the global fund is seven times more than grants. These findings indicate that all countries and governments have taken the TB eradication plan seriously and are employed towards achieving this important universal target.

**Fig. 3:**
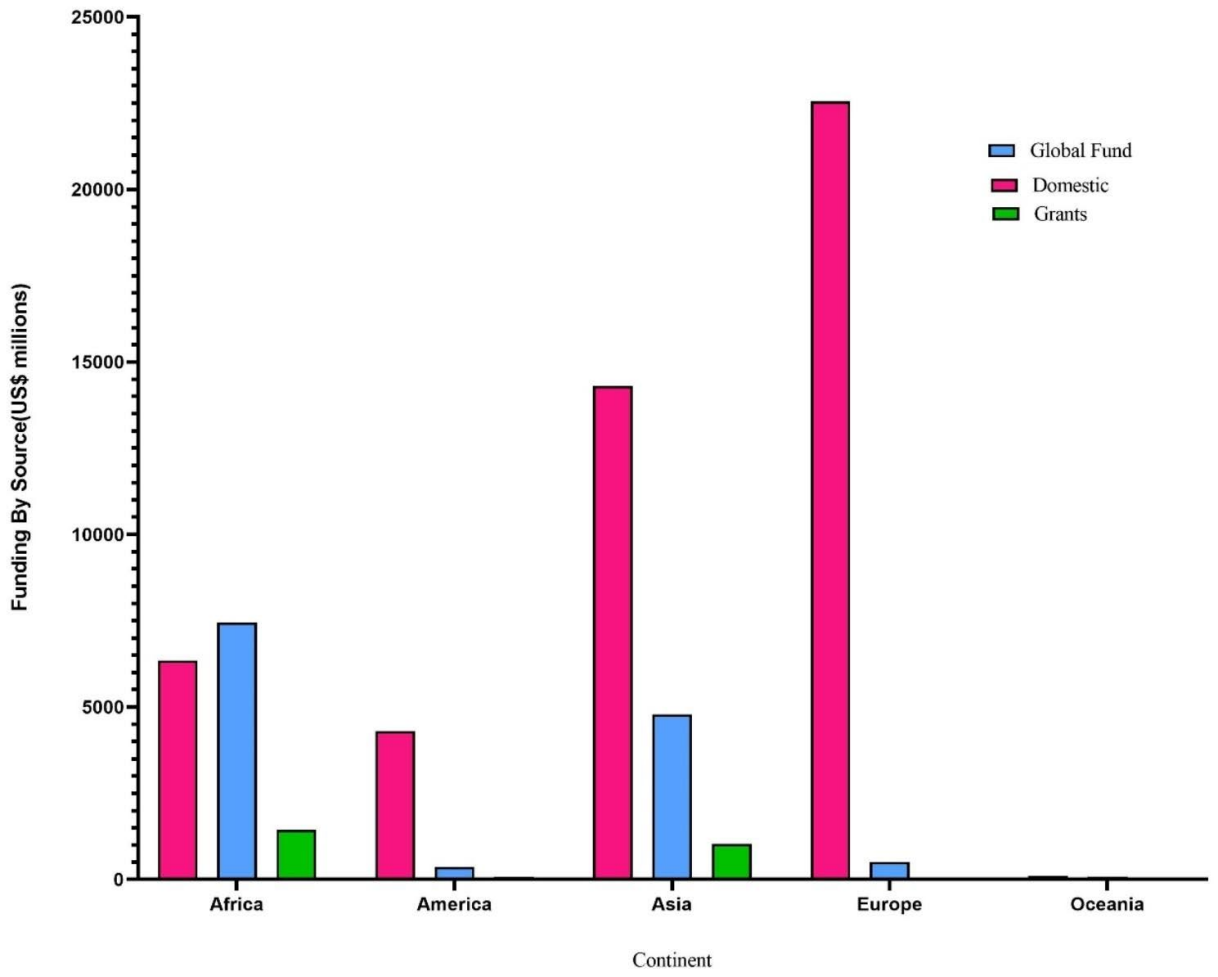
Analysis of funding by source (US $ million) by continent from 2006-2021base on the global TB report 2021

Another part of our analysis that analyzed the data by continent provided considerable information, shown in Fig. 3. In all continents, domestic funds are more than global funds and grants, except in Africa. In Africa, global funds are more than domestic funds and grants. This means that Africa has received more attention from global funds and has been more successful in receiving them than other continents. According to the reports of the WHO, 30 countries have been reported as high TB burden countries.

One of the reasons for the global attention to the Africa can be due to the presence of more than half of these 30 countries in the African continent. Perhaps it can be concluded that if the countries with high TB burden countries are given more attention and allocated more funds to these countries for TB diagnosis and treatment, the TB eradication plan will be pursued more successfully. On the other hand, it may effectively reduce the time to achieve this global goal, and tuberculosis will be eradicated in a shorter time.

### Tuberculosis financial profile by continent

Considering Fig. 3, the global funds and grants are the most in Africa and Asia and the least in Oceania during 2006–2021. Besides, Fig. 3 indicated that Europe dedicated the most domestic funds to TB eradication during 2006–2021. Furthermore, Oceania dedicates the least domestic funds to TB eradication. Overall, Europe, Asia, and Africa allocated the most funds, and Oceania and America allocated the least funds to the TB eradication plan during 2006–2021. This shows that the allocation of domestic funds, global funds, and grants for all continents has not been the same, and each continent has acted according to its special planning to eradicate TB.

### Tuberculosis financial profile by year and continent

**Fig. 4:**
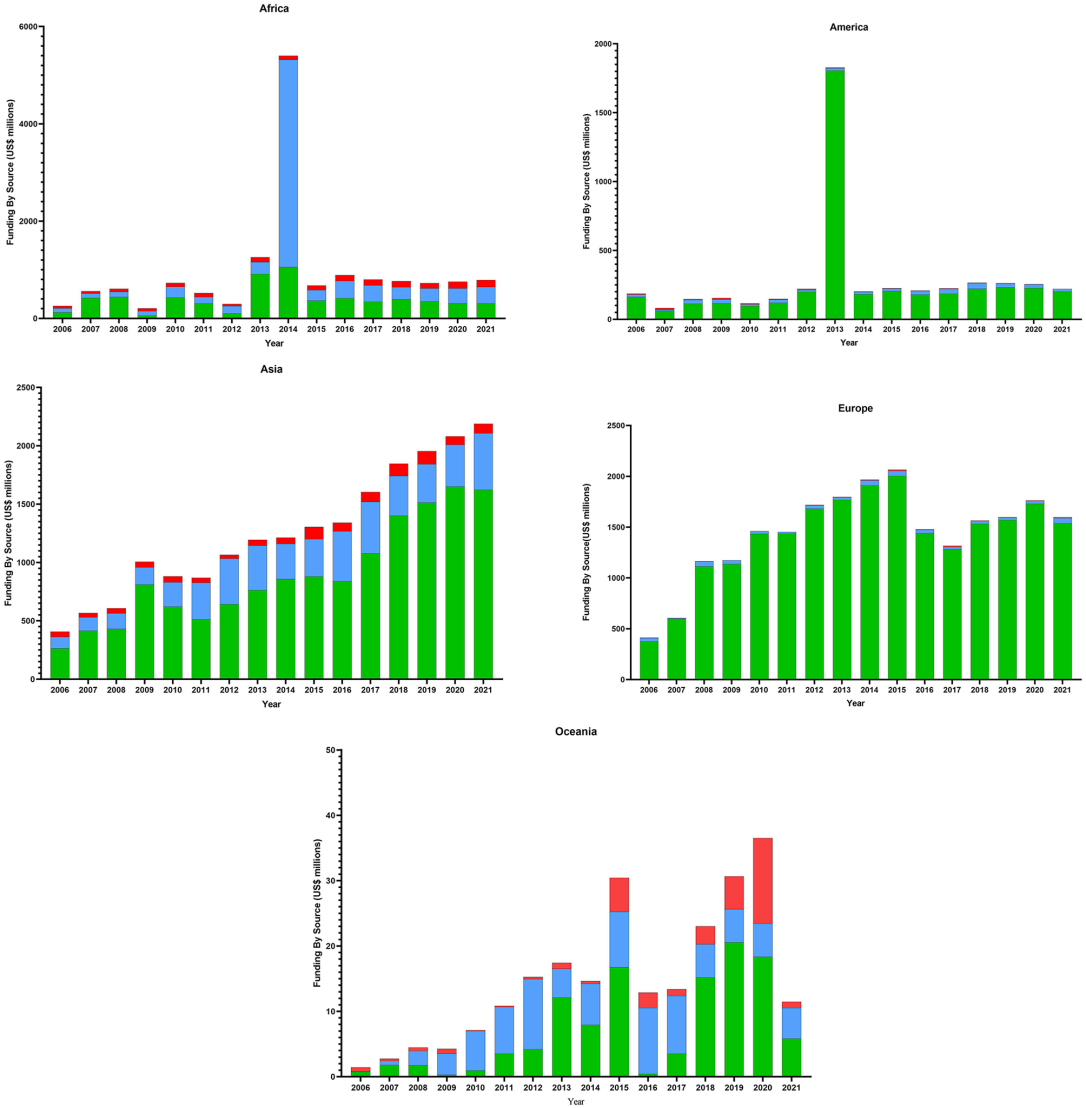
Analysis of funding by source (US $ million) from 2006-2021base on the global TB report 2021 for each continent. Green: domestic funds, blue: global funds, and red: grants

Interestingly the analysis for each continent illustrates considerable information to control and eradicate the TB plan (Fig. 4). In Africa, the most funds were allocated in 2014, 2013, and 2016, while the least was in 2009, 2006, and 2012. Domestic funds, global funds, and grants have a slow-rising slope. The domestic funds were the most in 2014, 2013, and 2008 and the least in 2009, 2012, and 2006. The most global funds were in 2014, 2016, and 2021 and the least were in 2006, 2007, and 2009. The most grants were in 2021, 2020, and 2018 and the least were in 2012, 2006, and 2007.

In America (Fig. 4), the most funds were allocated in 2013, 2018, and 2019 and the least were in 2007, 2010, and 2008. The grants and global funds have a very slow rise, while the domestic funds have a steeply rising slope. The domestic funds were the most in 2013, 2019, and 2020 and the least in 2007, 2010, and 2008. The most global funds were in 2018, 2017, and 2019 and the least were in 2007, 2010, and 2012. The most grants were in 2007, 2009, and 2012 and the least were in 2021, 2019, and 2018.

In Asia (Fig. 4), the most funds were allocated in 2021, 2020, and 2019 and the least were in 2006, 2007, and 2008. The increasing slope decreases domestic funds, global funds, and grants. The domestic funds have the steepest rising slope. The domestic funds were the most in 2020, 2021, and 2019 and the least in 2006, 2007, and 2008. The most global funds were in 2021, 2017, and 2016 and the least were in 2006, 2007, and 2008. The most grants were in 2019, 2018, and 2015 and the least were in 2012, 2007, and 2011.

In Europe (Fig. 4), the most funds were allocated in 2015, 2014, and 2013 and the least were in 2006, 2007, and 2008. The increasing slope for domestic funds was steep, while the slope for global funds and grants was very slow. The domestic funds were the most in 2015, 2014, and 2013 and the least in 2006, 2007, and 2008. The most global funds were in 2007, 2011, and 2017 and the least were in 2015, 2021, and 2014. The most grants were in 2015, 2017, and 2021 and the least were in 2011, 2010, and 2012.

In Oceania (Fig. 4), the most funds were allocated in 2020, 2019, and 2015 and the least were in 2006, 2007, and 2009. Domestic funds and grants have a steeply rising slope, whereas global funds have a slow slope. The domestic funds were the most in 2019, 2020, 2015 and the least in 2009, 2016, and 2006. The most global funds were in 2012, 2016, and 2017 and the least were in 2006, 2007, and 2008. The most grants were in 2020, 2015, and 2019 and the least were in 2010, 2011, and 2012.

### Tuberculosis financial profile by countries

**Fig. 5:**
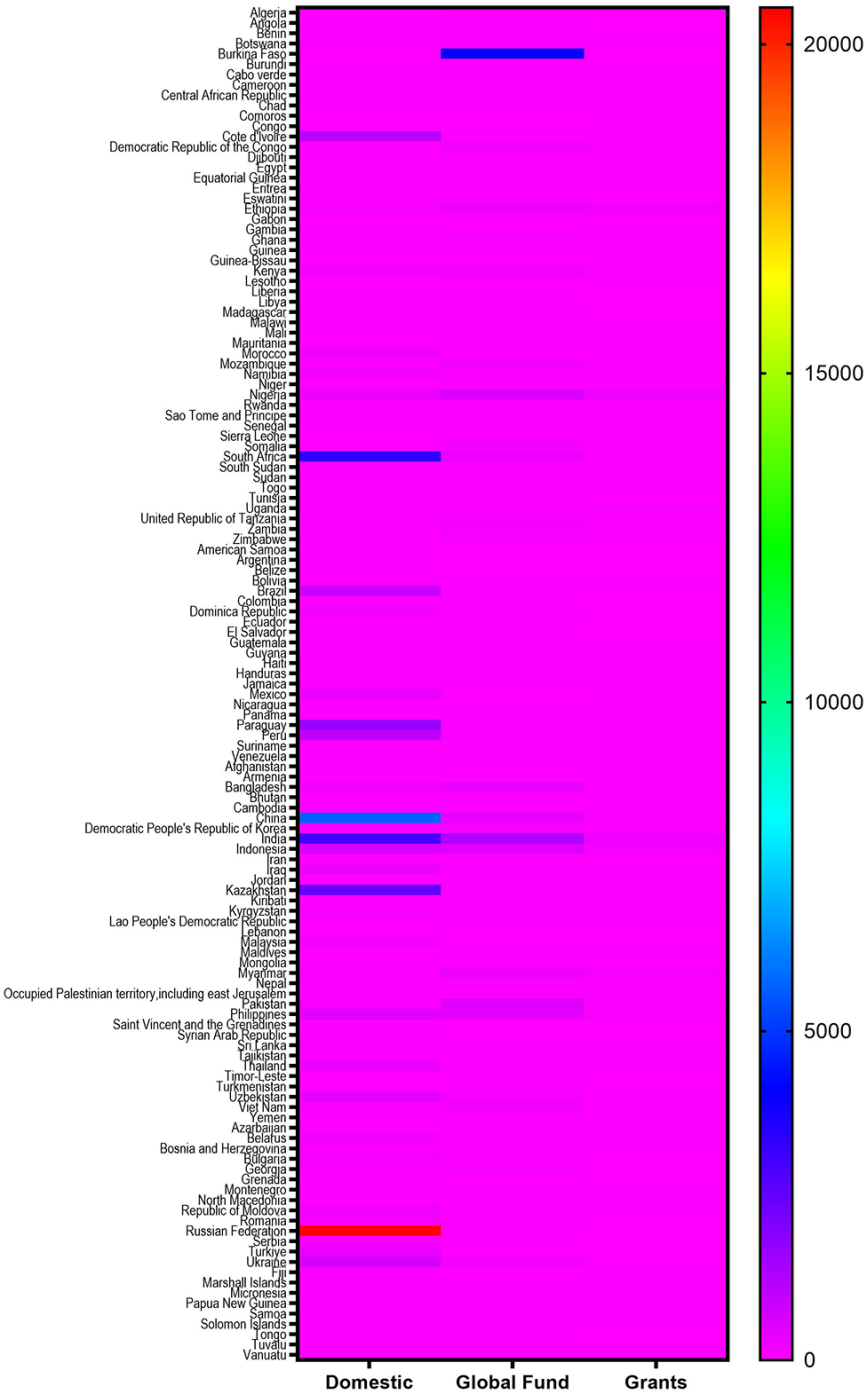
Heat map analysis of funding by source (US $ million) from 2006-2021base on the global TB report 2021 for each country

The 126 countries were examined for differences in domestic funds, global funds, and grants. Supplementary Table 1 is a condensed version of all country details. Figure 5 presents a heat map of the domestic funds, global funds, and grants to visualize these differences.

As Fig. 5 illustrates, during 2006–2021, Burkina Faso, Comoros, Somalia, and Turkmenistan have not indicated any domestic funds for the TB eradication plan. Grenada, Djibouti, Saint Vincent and the Grenadines, the Gambia, American Samoa, Tuvalu, Tongo, Montenegro, the Maldives, Samoa, and the Cabo Verde indicated the least domestic funds (< 1 US $ million) to TB eradication plan. The Russian Federation allocated the most domestic funds (> 20,000 US $ million) to the TB eradication plan, which was followed by China, South Africa, India, Kazakhstan, Paraguay, Cote d’Ivoire, and Peru (> 1,000 US $ million).

Additionally, Fig. 5 exemplifies that during 2006–2021, Comoros, Grenada, Saint Vincent and the Grenadines, American Samoa, Algeria, Argentina, Malaysia, Mexico, and Turkey have not received any global funds for the TB eradication plan. The Maldives, Samoa, Tongo, Jamaica, Cabo Verde, Tuvalu, Kiribati, and Micronesia received the least global funds (< 1 US $ million) for the TB eradication plan. Burkina Faso received the most domestic funds (> 4,000 US $ million) for the TB eradication plan, which is followed by India, Nigeria, Pakistan, and Indonesia (> 500 US $ million).

Besides, heat map analysis shows Grenada, Saint Vincent and the Grenadines, American Samoa, Algeria, Argentina, Malaysia, Turkey, Tongo, Libya, Belize, Turkmenistan, Iran, Tunisia, Serbia, Colombia, Ecuador, Syrian Arab Republic, Bulgaria, the Russian Federation, and Burkina Faso have not received any grants. Tuvalu, Jamaica, Samoa, Montenegro, Gambia, Bosnia and Herzegovina, the Maldives, and El Salvador received < 0.1 US $ million. India received the most grants (> 250 US $ million) for the TB eradication plan, which is followed by Nigeria, Ethiopia, Indonesia, South Africa, and Myanmar (> 100 US $ million).

## Conclusion

Analyzing available data can help authorities decide how to assign different sources of funds to different countries to eradicate TB as soon as possible. Hence, this study aimed to collect and analyze data from articles and databases. Numerous researchers in different parts of the world analyzed the available data to predict the TB eradication plan; nevertheless, no paper has been published that analyzed the financial profile of TB prevention, diagnosis, and treatment from 2006 to 2021worldwide. On average, 5107.475 US $ million per year from 2006 to 2021 have been allocated to the TB eradication plan.

The most assigned funds were in 2014 (8797.58 US $ million) and 2011 (7038.08 US $ million) worldwide, during 200–2021. The least allocated funds were also in 2006 (1263.54 US $ million), followed by 2007 (1815.97 US $ million). In 2019, when six of the largest continental budgets were allocated to the TB eradication program, and in 2007, ten of the lowest continental budgets were allocated to the TB eradication plan. Our obtained results show different continents earmarked wide-ranging funds for the mentioned plan in several years, Africa (the most: 2014, the least: 2009), America (the most: 2013, the least: 2007), Asia (the most: 2021, the least: 2006), Europe (the most: 2015, the least: 2006), and Oceania (the most: 2020, the least: 2006). Allocation of funds in different countries and proper planning for TB eradication has caused that in the last two decades, the slope of the confirmed cases and deaths graph line is negative, and the number of confirmed cases and deaths reported globally is decreasing. As a limitation, in the present study, our focus was on the financial profile of every country which could be affected by other factors such as population, climate, race, etc. In future studies, the other confounding variables should be considered as undeniable variables. In the future, additional analyses based on the updated data are essential to analyze the TB financial profile to eradicate TB in 2035 or sooner.

### Electronic supplementary material

Below is the link to the electronic supplementary material.

Additional File 1: Supplemntry Table 1. The detail of the funding source includes domestic, global, and grants (excluding global funds) in all country between 2006-2021

## Data Availability

The data used to support the findings of this study are included in the article and supplementary file.
